# Effect of disease duration on the association between C-reactive protein-albumin ratio and endoscopic activity in ulcerative colitis

**DOI:** 10.1186/s12876-022-02113-3

**Published:** 2022-01-30

**Authors:** Shinya Furukawa, Sen Yagi, Kana Shiraishi, Teruki Miyake, Kazuhiro Tange, Yu Hashimoto, Shogo Kitahata, Tomoe Kawamura, Tomoyuki Ninomiya, Kenichirou Mori, Seiyuu Suzuki, Naozumi Shibata, Hidehiro Murakami, Katsuhisa Ohashi, Aki Hasebe, Hideomi Tomida, Yasunori Yamamoto, Eiji Takeshita, Yoshio Ikeda, Yoichi Hiasa

**Affiliations:** 1 Health Services Center, Ehime University, Matsuyama, Ehime 790-8577 Japan; 2 Department of Internal Medicine, Saiseikai Matsuyama Hospital, Matsuyama, Ehime 791-8026 Japan; 3 Department of Gastroenterology and Metabology, Ehime University Graduate School of Medicine, Toon, Ehime 791-0295 Japan; 4 Department of Inflammatory Bowel Diseases and Therapeutics, Ehime University Graduate School of Medicine, Toon, Ehime 791-0295 Japan; 5 Department of Gastroenterology, Ehime Prefectural Central Hospital, Matsuyama, Ehime 790-0024 Japan; 6 Department of Internal Medicine, Iyo Hospital, Iyo, Ehime 799-3101 Japan; 7 Department of Gastroenterology, Sumitomo Besshi Hospital, Niihama, Ehime 792-8543 Japan; 8 Department of Gastroenterology, Ehime Prefectural Niihama Hospital, Niihama, Ehime 792-0042 Japan; 9 Ohashi Clinic Participate in Gastro-Enterology and Ano-Proctology, Niihama, Ehime 792-2856 Japan; 10 Department of Gastroenterology, Shikoku Cancer Center, Matsuyama, Ehime 791-0280 Japan; 11 Endoscopy Center, Ehime University Hospital, Toon, Ehime 791-0295 Japan

**Keywords:** Mucosal healing, Ulcerative colitis, Clinical remission, Albumin, CRP/albumin ratio

## Abstract

**Background:**

A simple serum biomarker for clinical outcome in patients with ulcerative colitis (UC) remains an unmet need. Some studies have shown an association between C-reactive protein (CRP)-albumin ratio (CAR) and prognosis in patients with inflammatory bowel disease (IBD), but evidence regarding the association between CAR and UC remains limited. We evaluated the association between CAR and clinical outcome in Japanese patients with UC.

**Methods:**

Subjects were 273 Japanese patients with UC. Clinical remission was defined as absence of both abnormally high stool frequency (< 3 per day) and rectal bleeding. Mucosal healing (MH) was defined as Mayo endoscopic subscore (MES) 0. Moderate to severe endoscopic activity was defined as MES 2–3. Subjects were divided according to CAR into tertiles (low, moderate, and high).

**Results:**

The proportions of patients with clinical remission, MH, and moderate to severe endoscopic activity were 57.9%, 26.0%, and 37.0%, respectively. High CAR was significantly positively associated with moderate to severe endoscopic activity but not MH or clinical remission after adjustment (adjusted odds ratio [OR] 2.18 [95% confidence interval (CI) (1.11–4.35)], *p* for trend 0.023), but only in patients with long disease duration (> 7 years) (adjusted OR 2.95 [95% CI (1.06–8.79)], *p* for trend 0.023). CAR was not associated with clinical remission or MH.

**Conclusions:**

CAR may be significantly positively associated with moderate to severe endoscopic activity but not clinical remission or MH in Japanese patients with UC. In patients with long UC duration, CAR might be a useful serum marker for disease activity.

## Background

Ulcerative colitis (UC), one of the inflammatory bowel diseases (IBD), is affecting growing numbers of patients worldwide [1]. While the clinical course of UC is characterized by repeated relapses and remissions [2], the majority of patients tend to have mild to moderate disease activity [3]. Yet 10–20% of patients have acute severe UC that persists after onset, and severe UC flare-up is associated with a 1% mortality rate [3]. The association between anti-tumor necrosis factor (TNF) α monoclonal antibody treatment and long-term colectomy rate among UC patients is still unclear [4].　Early detection of relapses, flares, changes in disease activity, and responses to treatments is key to recommending appropriate additional treatments, and thus to preventing complications and improving prognosis and quality of life [5, 6]. Despite many investigations into possible associations between various biomarkers and UC disease activity, the available serum biomarkers still have lower sensitivity and specificity for disease activity than fecal markers[7]. Accordingly, colonoscopy remains the gold standard for assessing UC disease activity. A serum biomarker with comparable sensitivity and specificity would allow us to assess disease activity more easily, more repeatably, and less invasively than we currently can.

Recently, some studies have reported an association between the C-reactive protein (CRP)-to-albumin ratio (CAR), a useful serum marker for several other diseases [8–12], and UC [13–16]. Evidence regarding the association between CAR and clinical outcomes in UC remains scarce, however. Here, we aimed to evaluate the association between CAR and clinical outcomes in UC as assessed in terms of clinical remission, mucosal healing and endoscopic disease severity and to explore whether duration of disease affects that association in Japanese patients with UC.

## Materials and methods

### Study population

The subjects of this study were patients with ulcerative colitis attending or hospitalized at the Department of Gastroenterology and Metabolism, Ehime University Graduate School of Medicine or affiliated hospitals in Ehime Prefecture from 2015 to 2019. All patients whose attending physicians were expected to be able to answer the self-administered questionnaire were included in the study. Most UC cases were diagnosed on the basis of endoscopic findings, which were additionally confirmed by radiographic and histological findings.

This study was planned in accordance with the Declaration of Helsinki, and the study protocol was reviewed and approved by the Institutional Review Board of Ehime University (Institutional approval number #1505011). Written informed consent was obtained from all enrolled patients by well-trained staff. The final analysis sample for this analysis consisted of 273 patients out of 387 UC patients enrolled in this cohort study, excluding 114 patients with incomplete data.

### Variables analysed

To obtain information on body mass index, endoscopic findings, disease extent, clinical remission, and the use of medication for UC, we examined patients’ medical records. Body mass index (BMI) was calculated as weight in kilograms divided by height in meters squared. Blood samples were taken in the morning after overnight fasting. Blood examination was performed when a colonoscopy was booked or when the colonoscopy was performed; there may have been an interval of up to two months between the blood examination and the colonoscopy.

### Assessment of clinical remission, endoscopic activity and MH

The definition of clinical remission was based on no rectal bleeding and no abnormally high stool frequency (cutoff frequency: three times per day). MH and moderate to severe endoscopic activity were assessed using the Mayo endoscopic subscore (MES) [17]. In this study, the definition of MH was MES 0; moderate and severe activity were defined as MES 2 and 3, respectively. Certified gastrointestinal endoscopists reported their endoscopic findings, and one endoscopist, who was blinded to the CAR level, was responsible for interpretation of the endoscopic findings.

### Statistical analysis

CARs were divided into categories by thirds based on the distribution of all subjects. These tertiles were used to group subjects into three categories: 1) low CAR, ≤ 0.012 (reference); 2) moderate CAR, 0.012–0.038; and 3) high CAR, ≥ 0.038. Multiple comparisons were analyzed using the Steel–Dwass test. The crude odds ratios (ORs) and their 95% confidence intervals (CIs) for clinical remission, MH, and moderate to severe endoscopic activity in relation to CAR were analyzed using logistic regression analysis. Age, gender, BMI, prednisolone use, anti-TNFα monoclonal antibody preparation, extent of disease (proctitis/non-proctitis), and disease duration were selected a priori as potential confounders. Multiple logistic regression analyses were used to adjust for potential confounders. Most of statistical analyses were performed using SAS software package version 9.4 (SAS Institute Inc., Cary, NC, USA). A receiver operating characteristic (ROC) curve, cutoff value of CAR (Youden’s index), sensitivity, and specificity were analyzed using JMP 14.2 software (SAS Institute Inc.).

## Results

**Fig. 1:**
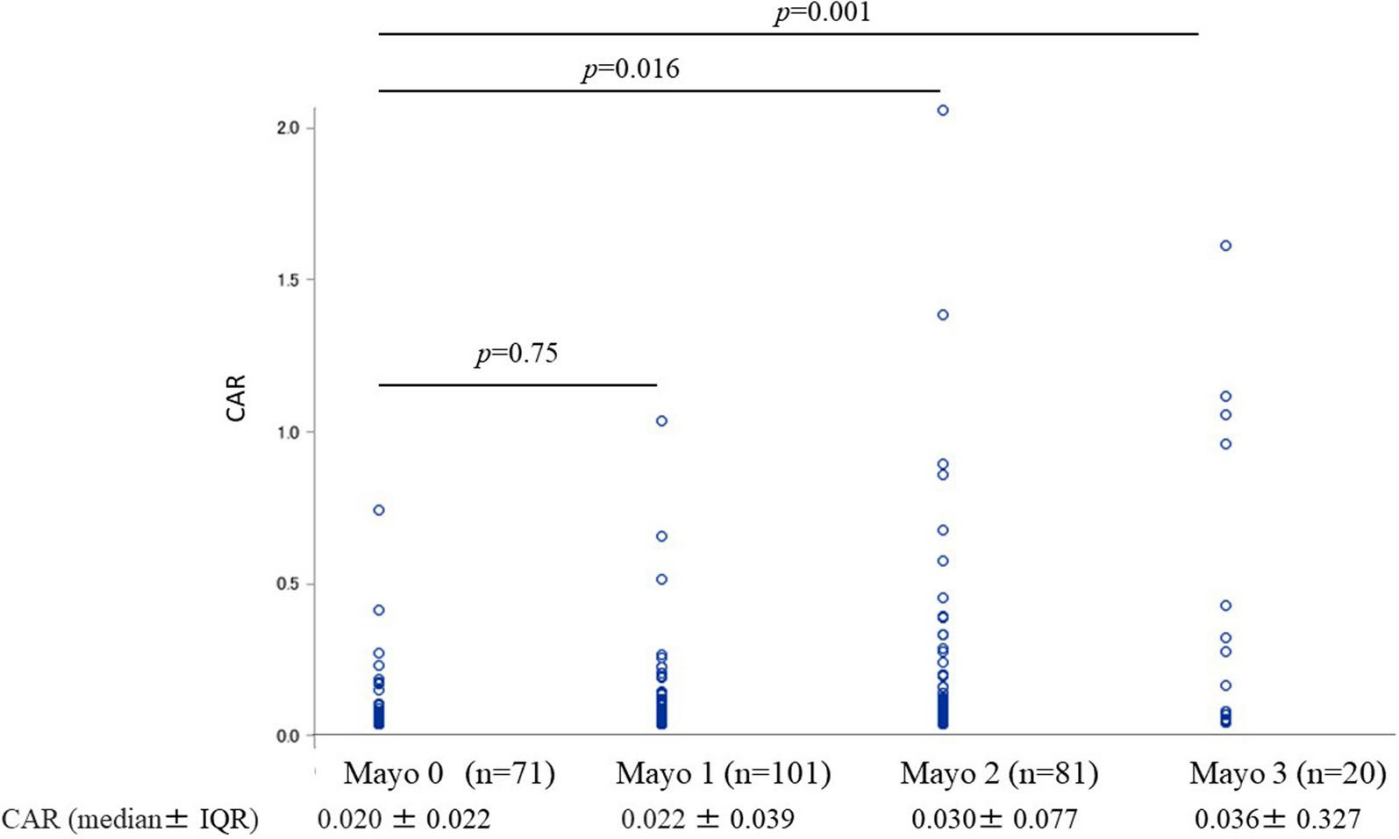
CAR value by Mayo score

Table 1 shows the characteristics of this cohort. The rates of clinical remission, MH, and moderate to severe endoscopic activity were 57.9%, 26.0%, and 37.0%, respectively. Prednisolone and TNF-α monoclonal antibody preparations were used by 21.6% and 6.2% of patients, respectively. Mean serum albumin, median CRP, and median CAR were 4.218 g/dl, 0.099 mg/dl and 0.022, respectively. The CAR value in MES 2 or 3 was significantly higher than that in MES 0 (Fig. 1). There was no significant difference in CAR values between MES2 and MES3.

**Table 1:** Clinical characteristics of 273 study participants

Variable	n (%)
Age, years, mean ± SD	51.0 ± 16.0
Male sex (%)	161 (59.0)
BMI, kg/m^2^	22.72 ± 4.62
Disease extent (pancolitis/left-sided/proctitis/others)	113/74/79/7
Disease duration, years, median ± IQR	7.0 ± 11.0
Medication	
5-aminosalicylates (%)	249 (91.2)
Prednisolone (%)	59 (21.6)
TNF-α monoclonal antibody (%)	17 (6.2)
Azathioprine (%)	41 (15.0)
Clinical remission, %	158 (57.9)
MES, mean ± SD	1.18 ± 0.91
MES 0, n (%)	71 (26.0)
MES 1, n (%)	101 (37.0)
MES 2, n (%)	81 (29.7)
MES 3, n (%)	20 (7.3)
Mucosal healing (MES = 0) (%)	71 (26.0)
Moderate to severe UC (MES 2 or MES 3)	101 (37.0)
Albumin, g/dl, mean ± SD	4.218 ± 0.43
CRP, mg/dl, median ± IQR	0.099 ± 0.23
CAR, median ± IQR	0.022 ± 0.053

SD, standard deviation; IQR, interquartile range; BMI, body mass index; TNF, tumor necrosis factor; MES, Mayo Endoscopic Subscore; CRP, C-reactive protein; CAR, C-reactive protein to albumin ratio

Table 2 shows the crude and adjusted ORs and 95%CIs for clinical remission, MH, and moderate to severe endoscopic activity in relation to CAR. High CAR was inversely associated with MH (crude OR 0.48 [95%CI: 0.23–0.97]); after adjustment for age, sex, BMI, prednisolone use, anti-TNFα monoclonal antibody preparation, disease extent (proctitis/non-proctitis), and disease duration, however, the inverse association between CAR and MH disappeared. A positive association between CAR and moderate to severe endoscopic activity (MES 2 or 3) was found (crude OR 2.39 [95%CI: 1.31–4.43]); after adjustment, this association was still significant (adjusted OR 2.18 [95%CI: 1.11–4.35], *p* for trend = 0.023).

**Table 2:** Crude and adjusted odds ratios and 95% confidence intervals for associations between clinical outcomes and CAR

Variable	Prevalence (%)	Crude OR (95%CI)	Adjusted OR (95%CI)
Clinical remission			
Low CAR	58/93 (62.4)	1.00	1.00
Moderate CAR	56/89 (62.9)	1.02 (0.56–1.87)	1.15 (0.59–2.25)
High CAR	44/91 (48.4)	0.57 (0.31–1.01)	0.58 (0.30–1.15)
*p* for trend			0.12
Mucosal healing (MES < 1)			
Low CAR	27/93 (29.0)	1.00	1.00
Moderate CAR	29/89 (32.6)	1.18 (0.63–2.23)	1.35 (0.69–2.68)
High CAR	15/91 (16.5)	0.48 (0.23–0.97)	0.49 (0.22–1.07)
*p* for trend			0.10
Moderate to severe activity (MES 2 or 3)			
Low CAR	27/93 (29.0)	1.00	1.00
Moderate CAR	29/89 (32.6)	1.18 (0.63–2.23)	1.12 (0.57–2.20)
High CAR	45/91 (49.5)	2.39 (1.31–4.43)	2.18 (1.11–4.35)
*p* for trend			0.023

Odds ratios were adjusted for age, sex, BMI, use of prednisolone, use of TNF-α monoclonal antibody, C-reactive protein, disease extent and disease duration. OR, odds ratio; CI, confidence interval; CAR, C-reactive protein to albumin ratio; MES, Mayo Endoscopic Subscore

The association between CAR and clinical outcomes in patient groups classified according to disease duration is shown in Table 3. In the short-duration group (≤ 7 years), high CAR was positively associated with moderate to severe endoscopic activity (crude OR 2.41 [95% CI: 1.08–5.51]; after adjustment, however, this association completely disappeared. In the long-duration group (> 7 years), in contrast, high CAR was independently associated with moderate to severe endoscopic activity even after adjustment (adjusted OR 2.95 [95% CI: 1.06–8.79], *p* for trend = 0.032). CAR was not associated with clinical remission or MH regardless of disease duration.

**Table 3:** Crude and adjusted odds ratios and 95% confidence intervals for associations between clinical outcomes and CAR according to duration of UC. Short duration (≤ 7 years) (n = 143)

Variable	Prevalence (%)	Crude OR (95%CI)	Adjusted OR (95%CI)
**Short duration (≤ 7 years) (n = 143)**			
Clinical remission			
Low CAR	29/53 (54.7)	1.00	1.00
Moderate CAR	23/43 (53.5)	0.95 (0.42–2.14)	0.93 (0.38–2.27)
High CAR	20/47 (42.6)	0.61 (0.28–1.35)	0.61 (0.24–1.53)
*p* for trend			0.30
Mucosal healing (MES < 1)			
Low CAR	12/43 (22.6)	1.00	1.00
Moderate CAR	12/43 (27.9)	1.32 (0.52–3.37)	1.51 (0.56–4.09)
High CAR	6/47 (12.8)	0.50 (0.16–1.42)	0.54 (0.16–1.74)
*p* for trend			0.39
Moderate to severe activity (MES 2 or 3)			
Low CAR	17/53 (32.1)	1.00	1.00
Moderate CAR	17/43 (39.5)	1.39 (0.60–3.23)	1.36 (0.53–3.48)
High CAR	25/47 (53.2)	2.41 (1.08–5.51)	1.56 (0.62–3.95)
*p* for trend			0.35
**Long duration (> 7 years) (n = 130)**			
Clinical remission			
Low CAR	29/40 (72.5)	1.00	1.00
Moderate CAR	33/46 (71.7)	0.96 (0.37–2.48)	1.38 (0.49–3.99)
High CAR	24/44 (54.6)	0.46 (0.18–1.12)	0.56 (0.20–1.56)
*p* for trend			0.24
Mucosal healing (MES < 1)			
Low CAR	15/40 (37.5)	1.00	1.00
Moderate CAR	17/46 (37.0)	0.98 (0.41–2.36)	1.23 (0.47–3.25)
High CAR	9/44 (20.5)	0.43 (0.16–1.12)	0.44 (0.14–1.29)
*p* for trend			0.15
Moderate to severe activity (MES 2 or 3)			
Low CAR	10/40 (25.0)	1.00	1.00
Moderate CAR	12/46 (26.1)	1.06 (0.40–2.85)	0.81 (0.27–2.36)
High CAR	20/44 (45.5)	2.50 (1.004–6.53)	2.95 (1.06–8.79)
*p* for trend			0.032

Odds ratios were adjusted for age, sex, BMI, use of prednisolone, use of TNF-α monoclonal antibody, C-reactive protein, and disease extent. OR, odds ratio; CI, confidence interval; UC, ulcerative colitis; CAR, C-reactive protein to albumin ratio; MES, Mayo Endoscopic Subscore

**Fig. 2:**
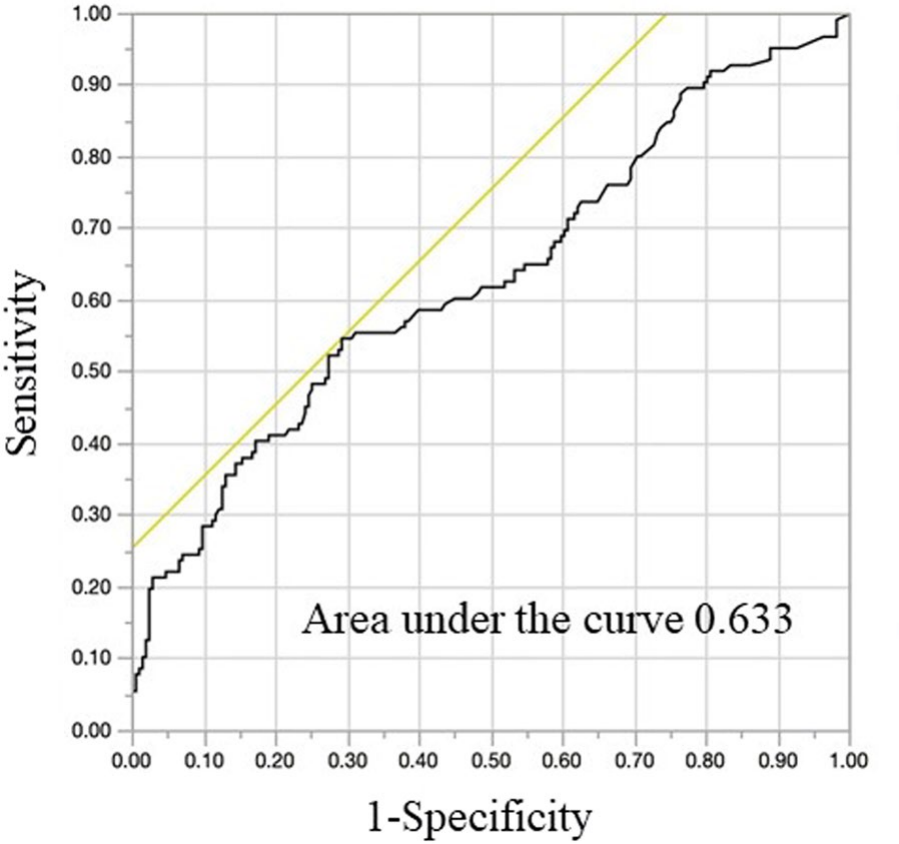
Receiver operating characteristic curve of CAR as a marker for MES 2 and 3. The receiver operating characteristic (ROC) curve of CAR as a marker for moderate-severe stage had an area under the curve of 0.633. When the cut-off CAR level was set to 0.030, the sensitivity and specificity for MH were 54.8% and 70.8%, respectively

The predictive value of CAR for moderate to severe disease activity was investigated through ROC curve analysis (Fig. 2). The area under the curve was 0.633. The sensitivity and specificity for moderate to severe endoscopic activity (with a CAR cut-off value of 0.030) were 54.8% and 70.8%, respectively. In sensitive analysis, the CAR value in pancolitis (0.024 ± 0.120) was higher than that in proctitis (0.014 ± 0.020). Serum albumin (mean ± SD) and CRP (median ± IQR) value in low CAR, moderate CAR, and high CAR were 4.32 ± 0.34 g/dl, 0.03 ± 0.02 mg/dl; 4.29 ± 0.36 g/dl, 0.10 ± 0.03 mg/dl; and 4.03 ± 0.52 g/dl, 0.45 ± 0.72 mg/dl, respectively.

## Discussion

The present study demonstrated that CAR was independently positively associated with moderate to severe endoscopic activity in patients with UC. No association was found between CAR and other clinical outcomes including MH and clinical remission.

Several previous studies have explored the association between CAR and clinical outcomes in patients with UC. A Turkish study of 149 patients with UC found a relationship between CAR and clinical disease activity whereby CAR value was associated with the difference between moderate and severe disease activity [13].In a Chinese study of 876 patients with IBD, including 275 patients with UC, CAR was highly useful for identifying patients with active-stage IBD [14]. In an Irish study of 124 patients with acute severe UC, CAR was significantly higher in steroid-refractory patients than in steroid-responsive patients [15]. In an Australian retrospective study of steroid-refractory UC patients, CAR was positively associated with colectomy after infliximab [16]. Taking these results together, CAR is positively associated with clinical activity, endoscopic activity, and poor response to medication in UC patients. The results of the present study are consistent with these previous findings indicating an association between CAR and clinical outcomes.

The Selecting Therapeutic Targets in Inflammatory Bowel Disease (STRIDE) study recommended treat-to-target strategies for patients with IBD [18, 19]. While maintaining MH remains the larger goal, the normalization of serum and fecal biomarkers has been identified as a short-term target. The development of an easy, rapid, repeatable, and affordable serum marker for MH is one of the unmet needs related to maintaining MH in the clinical setting. Several novel biomarkers for MH have recently been reported, including the CAR as well as serum globulin, serum indirect bilirubin, neutrophil/lymphocyte ratio, albumin/platelet ratio, and platelet/lymphocyte ratio [20–23]. Our results indicate that CAR should be used as serum marker for disease activity and poor response to treatment but not for MH.

Only a few studies have explored the influence of disease duration on biomarker accuracy in patients with IBD. In patients with Crohn’s disease, disease duration did not affect the utility of fecal calprotectin as a biomarker [24]. In patients with UC, on the other hand, the immunochemical fecal occult blood test as a biomarker for MH is more accurate in patients with disease duration < 4 years [25]. Similarly, serum albumin was found to be a useful marker for MH in patients with shorter UC duration but not in those with longer UC duration [26]. In the present study, CAR was associated with disease activity only in UC patients with longer disease duration but not in those with shorter disease duration. It remains uncertain why disease duration affects the association between CAR and disease activity in patients with UC. Further research regarding the association between disease duration and serum biomarker accuracy in patients with UC is warranted.

Although the mechanism underlying the link between CAR and disease activity remains unclear, there are several biologically plausible possibilities. Both CRP and albumin are widely used as acute inflammatory markers in clinical settings, and albumin is also used as a marker of malnutrition. During inflammation, cytokines such as TNF α and IL-6 may suppress albumin synthesis in the liver [27]. Chronic and severe inflammation leads to hypercytokinemia, which can lead to malnutrition [28]. It is possible that CAR is related to severe and/or chronic inflammation but not to mild inflammation. This would explain why CAR is associated with disease activity only in patients with long disease duration.

There are several limitations to this study. First of all, it was a cross-sectional study. Therefore, causality between CAR and MH needs to be confirmed by conducting longitudinal and interventional studies. Secondly, many of the patients in this cohort were on long-term treatment and had high clinical remission. Long-term treatment might affect the association between CAR and endoscopic activity. Third, fecal calprotectin is recognized as a reliable marker of MH, however, the calprotectin data was missing in this cohort. Fourth, other clinical scores, including the Clinical Activity Index, were missing in this cohort. Finally, selection bias may have affected the results. The characteristics of this cohort might be different those of Japanese ulcerative colitis. However, the sex ratio, median age (years), and frequency of anti-TNFα monoclonal antibody use (63.9%, 44.0, and 9.0%, respectively) in the Japanese national study were similar to those in this study (57.9%, 48.0, and 6.2%, respectively) [29].

## Conclusions

In Japanese patients with UC, CAR might be significantly positively associated with endoscopic activity. No association between CAR and MH was found in this cohort. In clinical settings, CAR can be used as a marker of disease activity but should not be used as a marker of remission, including MH, in patients with long UC duration.

## Data Availability

The datasets used and/or analyzed during the current study are available from the corresponding author on reasonable request.

## Abbreviations

Alb: Albumin
BMI: Body mass index
CAR: C-reactive protein/albumin ratio
CRP: C-reactive protein
MES: Mayo endoscopic subscore
MH: Mucosal healing
ROC: Receiver operating characteristic
STRIDE: Selecting Therapeutic Targets in Inflammatory Bowel Disease
TNF: Tumor necrosis factor
UC: Ulcerative colitis
